# Healthcare Access in Medically Underserved Areas During the COVID-19 Era: An International Medical Graduate Perspective From a Rural State

**DOI:** 10.7759/cureus.12254

**Published:** 2020-12-24

**Authors:** Srikrishna V Malayala, Deepa Vasireddy, Renuka Ananth Kalyan Kadali, Ram Sanjeev Alur, Kiran Koushik

**Affiliations:** 1 Internal Medicine, Temple University Hospital, Philadelphia, USA; 2 Pediatrics, Pediatric Group of Acadiana, Lafayette, USA; 3 Internal Medicine, Central Harnett Hospital, Lillington, USA; 4 Internal Medicine, Marion Veterans Affairs Medical Center, Marion, USA; 5 Medicine, Piedmont Medical Center, Rock Hill, USA

**Keywords:** public health policy, epidemiology and public health, rural areas, internal medicine in rural areas, immigration policy, imgs, covid 19, covid-19 outbreak, coronavirus disease (covid-19), physician shortage

## Abstract

Background

Physician shortage and healthcare access are serious issues in rural states like Kentucky and further worsened during the coronavirus disease 2019 (COVID-19) pandemic. International Medical Graduates (IMGs) serve the underserved communities of Kentucky to fill in the physician gap. However, uncertainties surrounding immigration policies added significant challenges to physicians and the rural communities served by them during the pandemic.

Methods

A survey was created using the data collection platform “SurveyMonkey” and sent to IMG physicians practicing on a visa to understand their role and their immigration-related challenges. Only the physicians practicing in Kentucky were included in this study.

Results

It was found that 84% practice in primary care specialties like internal medicine, pediatrics, or family medicine, 92.9% practice in Medically Underserved Areas or Health Professional Shortage Areas, and 71.4% practice in rural settings. Also, 61.5% practice in a “frontline” COVID-19 specialty and 92.3% were involved in direct care of COVID-19 infected or suspected patients. Of the physicians, 88.5% were in an “immigration backlog”; 92.6% of them were the primary visa holders of their families and 88.9% expressed concern that their families face hardship if they have a disability during the pandemic. It was reported by 92.3% of them that visa-related restrictions limited them from providing additional coverage in these places.

Conclusions

Lack of physician access is a critical issue facing many rural states in America like Kentucky, and IMG physicians play a valuable role in taking care of this underserved population and fighting the COVID-19 pandemic. The challenges surrounding the immigration backlog are contributing to significant hardships and remain a hurdle to expand healthcare access to the rural and medically underserved communities.

## Introduction

Kentucky, officially the Commonwealth of Kentucky, is a rural state located in the Southern United States [1]. Like many other rural states in the US, the healthcare in the state of Kentucky is affected by a severe physician shortage owing to multiple factors [2]. Even prior to the pandemic, to maintain current rates of utilization, Kentucky will need a 24% increase compared to the state's current Primary Care Physician (PCP) workforce. The main reasons for Kentucky’s imminent need for PCPs include increased utilization due to aging, population growth, and a greater insured population following the Affordable Care Act (ACA) [3]. Along with physician shortage, other chronic healthcare issues challenging the state are rural hospital closure, cancer mortality, substance abuse, and mental health disorders [4-7]. Furthermore, the coronavirus disease 2019 (COVID-19) pandemic further dampened the healthcare in the state, leading to 213,000 people infected and 2,359 dead as of December 9, 2020 [8].

International Medical Graduates (IMGs) have been a longstanding solution to the physician shortage in Kentucky, constituting upto 21% of the active physician workforce in the state, with 80% of these IMG physicians being in primary care specialties [9]. Since the onset of the pandemic, IMG physicians are directly involved in patient care of COVID-19 patients all across the country, and more so in rural states like Kentucky. However, many policies surrounding the US-trained and licensed IMG physicians posed significant challenges to the physicians at their personal and professional fronts and limited their ability to fight the pandemic efficiently. One such significant reason was the “immigration backlog.” With the immigration being restricted to only 7% of individuals from each country, physicians of large population countries (like India and China) are stuck in an immigration backlog, causing years and sometimes decades of wait time to obtain a permanent residency [10].

The objective of the study is to study the demographics of the IMGs working in the state of Kentucky before and during COVID-19 pandemic, their contribution to COVID-19 related patient care, and the challenges they are experiencing due to their temporary immigration status.

## Materials and methods

A cross-sectional survey was posted on multiple social media platforms that are pertinent to the IMG physicians. The physician members of these social media pages were pre-screened based on their NPI (National Provider Identifier) numbers, and type and location of practice. All these physicians were either practicing physicians, residents and fellows in training, or prospective residents. The survey was designed and circulated using SurveyMonkey [11].

We used two screening questions in the survey to screen for the appropriate sample. The first question, “Are you an IMG practicing in the US?”, excluded all the non-IMG respondents. The second question “Are you practicing in the US on a visa?”, excluded the US citizens and permanent residents from participating in the survey. We asked for their state of practice and included the physicians practicing only in the state of Kentucky in the final sample.

Apart from the basic demographics such as age, state of practice, specialty and subspecialty, and board certification status, the details of these physicians pertinent to their and their family’s immigration status (current visa status, approval for permanent residency in the US, dependent visa status of their immediate family members, and status of children under 21 who do not have a US citizenship status) were analyzed.

Their involvement in the COVID-19 related direct and indirect patient care (working in rural versus urban settings-medically underserved areas, direct patient care of COVID-19 patients, “frontline caregiver” specialty, COVID-19 related administrative work) was identified. COVID-19 exposure related direct effects to the physicians, such as being positive for COVID-19, getting quarantined due to the exposure, and losing workdays due to the sickness, were reviewed. At the same time, indirect effects of the pandemic, such as losing days of work, taking a “pay cut”, and employment termination, were analyzed. The potential effects on their immigration status were studied (risk of families facing hardship in case of the physician’s disability, their risk of deportation in case of physician’s death and disability, limitation to work in “hot spots" or offer “moonlighting coverage” and additional telemedicine services due to their visa status).

SPSS Version 26 (IBM Corp., Armonk, NY, USA) was used to refine the data, analyze the data, and study the final sample.

## Results

From the final national sample, a total of 75 physicians were identified to be from Kentucky. Out of this total sample who acknowledged the survey, some identified themselves as non-IMGs and few of them already acquired permanent residency or US citizenship status, and hence they could not advance further in the study and were excluded. Furthermore, respondents with missing or unavailable data were also excluded. The final sample had 58 physicians from the state of Kentucky responding to the survey.

Demographics and patient care characteristics of the IMGs in the COVID-19 pandemic

The physician’s mean age was 34.8 years, with a standard deviation of 5.2 years. Majority of physicians practice internal medicine (67.9%) followed by neurology, pediatrics, family medicine, and psychiatry. Within Internal medicine, the most commonly practiced subspecialty was primary care (hospital medicine, general internist, or a combined track), followed by endocrinology, hematology, and gastroenterology in that order. All (100%) of these physicians are board-certified in their respective specialties; 92.9% of these physicians practice in a Medically Underserved Area (MUA) or Health Professional Shortage Areas (HPSA) and 71.4% of them were practicing in a rural setting. Around 61.5% of the physicians practice in a “frontline” COVID-19 pandemic specialty and 92.3% of them were involved in direct care of COVID-19 infected or suspected patients. Almost 57.7% of them are involved in administrative responsibilities of the COVID-19 preparedness in their hospitals, such as involvement in the command center and designing protocols (Table 1).

**Table 1:** Baseline demographics and patient care characteristics of IMGs practicing in Kentucky during the COVID-19 pandemic MUA, Medically Underserved Area; HPSA, Health Professional Shortage Area; IMG, International Medical Graduates

Characteristics	Response
Age (year), mean (SD)	34.9 (5.15)
Primary specialty, no. (%) (n=58)	
Internal medicine	39 (67.9)
Pediatrics	4 (7.1)
Family medicine	4 (7.1)
Neurology	9 (14.3)
Pathology	2 (3.6)
Board certification status, no. (%) (n=58)	58 (100%)
Practice in an MUA or HPSA, no. (%) (n=58)	54(92.9)
Practice in a rural setting, no. (%) (n=58)	41 (71.4)
Involved in direct patient care of COVID-19 positive or suspected patients, no. (%) (n=58)	53 (92.9)
Practicing in a “frontline” COVID-19 related specialty, no. (%) (n=58)	36 (61.5)
Administrative responsibilities for COVID-19 pandemic preparedness (incident commander etc.), no. (%) (n=58)	33 (57.7)

Immigration characteristics of the IMG physicians serving the pandemic

Table 2 explains the visa and immigration status of the physicians; 92.8% of them are practicing in an H1b status, either with or without a J1 waiver requirement. Around 64.3% of them have their immigration petition for permanent residency approved, but most of the physicians in this sample (88.5%) were in an “immigration backlog” owing to their country of origin. Also, 42.9% of the physicians had an immediate family member like a spouse or a child on a dependent visa and 92.6% of them were the primary visa holders of their families. A substantial number of physicians (7.1%) had a child who could “age out”, i.e., a child under 21 years of age and not born in the US.

**Table 2:** Immigration characteristics of the IMGs in the COVID-19 pandemic IMG, International Medical Graduate

Characteristics	Response
Current visa status, no. (%) (n=58)	
H1b (not requiring J1 waiver requirement)	14 (25.0)
H1b (completed J1 waiver requirement)	16 (28.6)
H1b (currently on a J1 waiver program)	21 (39.3)
Others	7 (7.2)
Have an approved petition for permanent residency (I-140), no. (%)	37 (64.3)
Have an immediate family member on a dependent visa, no. (%)	25 (42.9)
Have a child under 21 years and not a US citizen, no. (%)	7 (7.1)
Primary visa holder of the family, no. (%)	53 (92.6)
Immigration backlog based on country of origin, no. (%)	
No backlog	6 (11.5)
Extreme backlog (50-150 years)	52 (88.5)

Patient care and immigration-related challenges faced by the physicians in the pandemic

Kentucky IMG physicians serving the COVID-19 patients were not immune to the exposure from the COVID-19 infection. Almost 42.3% of the physicians were going through a loss or decrease in pay due to the pandemic-related issues, and a few of them (3.8%) had to lose workdays due to COVID exposure (Table 3).

**Table 3:** Practice- and immigration-related challenges of IMGs in the COVID-19 pandemic USCIS, U.S. Citizenship and Immigration Services; IMG, International Medical Graduate

Characteristics	Response
Practice-related challenges	
Lost workdays/shifts due to COVID-19 exposure or infection, no. (%) (n=58)	2 (3.8)
Taking a pay cut/loss of revenue due to the pandemic related issues, no. (%) (n=58)	24 (42.3)
Immigration challenges	
Will your family face hardship due to your unemployment or disability? no. (%) (n=58)	51 (88.9)
Is your family at risk of deportation in case of your death or disability? no. (%) (n=58)	38 (66.7)
Does suspension of premium visa processing by USCIS affect your stay or work in the US? no. (%) (n=58)	34 (59.3)
Do visa restrictions limit you from providing additional coverage in places of need during COVID-19 pandemic? no. (%) (n=58)	53 (92.3)
Do visa work restrictions limit you from providing telemedicine services to patients? no. (%) (n=58)	31 (53.8)
Did a recruiter or a hospital contact you to work at a site, city, or state due to staffing shortages during the COVID-19 pandemic? no. (%) (n=58)	29 (50)
Do you think a permanent residency status will help resolve most of the professional and personal concerns mentioned earlier? no. (%) (n=58)	51 (88.5)

Table 3 also illustrates the immigration-related challenges of the IMG physicians during the pandemic. Around 88.9% expressed concern that their families might face hardship in case of unemployment or disability during the pandemic, and 66.7 % of them were worried that their families are at risk of deportation in light of the primary visa holder’s disability or death. Furthermore, when U.S. Citizenship and Immigration Services (USCIS) suspended premium visa processing services in March 2020, 59.3% of the physicians felt that this could affect their stay or work in the US. When asked to rate on a scale of 1 to 4 asking what was most concerning to them in the COVID-19 pandemic, they reported risk of getting infected, risk of family deportation, risk of losing their job, and risk of facing financial hardship in that particular order. Around 88.5% of the physicians did express that a permanent residency status will solve most of their concerns and challenges.

During the pandemic, almost 50% of these physicians were approached by a recruiter or a hospital to provide part-time services in COVID-19 designated hot spots. However, 92.3% of them reported that visa-related restrictions limited them from providing additional coverage in these places. Work visa restrictions also limited 53.8% of them from providing telemedicine services.

## Discussion

**Figure 1:**
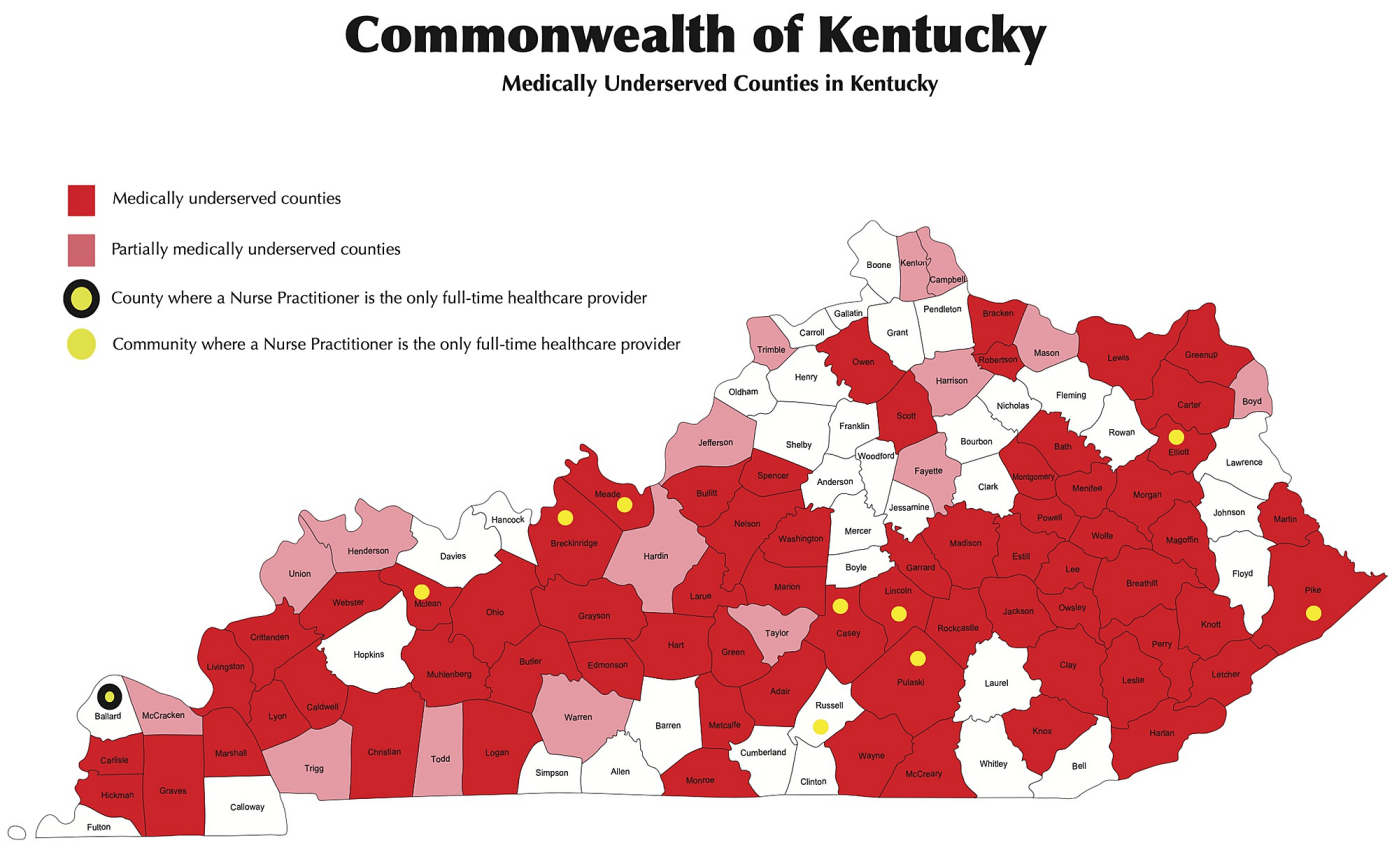
Medically Underserved Areas in the state of Kentucky

Kentucky is a prime example of a rural state in midwestern America [12]. Around 40% of Kentuckians live in rural areas, and only 17% of PCPs practice in rural areas (Figure 1).

Rural hospital closure has been a problem affecting the rural communities in the state and across the country. Nationwide, 113 hospitals have closed since 2010 and five hospitals have closed in Kentucky since 2009, four of them since 2014 [4]. Almost 65 of Kentucky’s 129 hospitals are rural and almost 35 of them are in poor financial health, and one-fourth of the rural hospitals in the state are at risk of closure [4,12]. Kentucky is also considered the nation’s “cancer capital”. Cancer mortality rate in Kentucky is 17% more than the national average [5]. The estimated cancer burden on the state was 3.8 billion dollars in 2020 [5]. Around 2.9 million people in Kentucky have at least one chronic disease, and 1.3 million have two or more chronic diseases; 53.5% of patients with mental health could not have access to treatment. Kentucky has the highest incidence of COPD in the country (one in every 10 residents) [6,7].

Along with the burden of chronic medical problems, physician shortage has always burdened the state, especially the rural parts. Kentucky ranks 40th in the number of PCPs per 100,000 population; 69 counties have 10 or less number of PCPs per county [2,12].

IMGs have been the bridge for rural healthcare needs and physician shortage in the US [13]. Among a few other visas, most of the IMGs use J1 exchange visas to complete their residencies in the US, with the expectation that they will return to their home countries and spend at least an aggregate of two years. Given the resource-intensive process of training a physician, there are several legislative programs in place, such as the Appalachian Regional Commission (ARC) program, HHS Visitor Exchange Program, Delta Regional Authority (DRA) program, and the Conrad J-1 Waiver 30 Program, with the primary purpose of retaining these assets to serve areas of the US with inadequate physician access [14]. Over the last decade or so, the state of Kentucky has used all the programs to its full capacity to keep up with the healthcare needs of the state. As we can see the results from our study, 92.9 % of the survey respondents are practicing in either an MUA or HPSA in Kentucky, and 71.4% of them were practicing in a rural setting. Of the physicians in our sample, 67.9% were providing internal medicine services and 82% of them were practicing in a primary care specialty like internal medicine, pediatrics, family medicine, and psychiatry.

After completion of their training, they become eligible to apply for a permanent resident status in the US, often colloquially referred to as a “green card” [15,16]. However, due to the limited annual quota of green cards, which are allocated based on the country of birth of the applicant, the wait times can vary widely among applicants. Due to the sheer number of applicants from countries like India and China, expectedly higher from the two most populous countries in the world, these applicants have the longest wait times before they become eligible to adjust their status to that of a permanent resident [10]. More so than any other state, this immigration backlog might hamper the healthcare needs of Kentucky as it is heavily dependent on the IMGs delivering frontline and primary healthcare. While 92.8% of the physicians in our sample were on H1b status, 64.3% of them already had their immigration petition for permanent residency approved and as much as 88.5% were in an “immigration backlog” as regulated by their country of origin [17].

**Figure 2:**
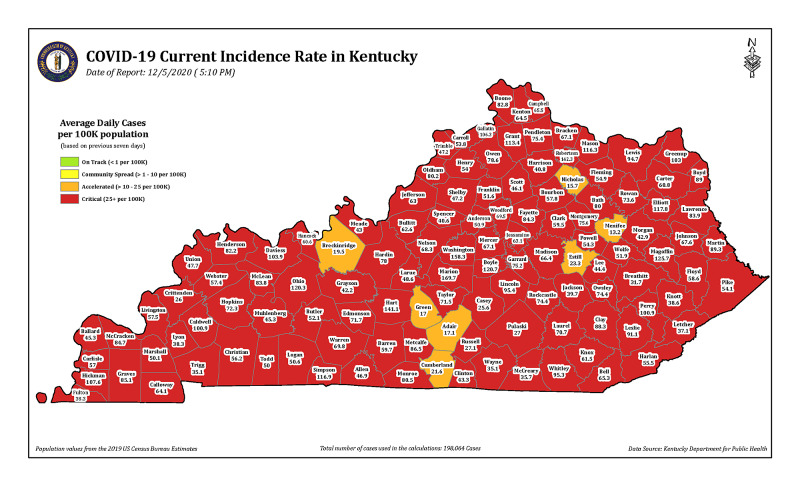
Current incidence of COVID-19 cases in Kentucky (as of December 9,2020) The seven-day incidence of the number of COVID-19 cases in Kentucky is calculated by taking the total number of unique cases in each county over the past seven days, divided by 7 to get a daily average, divided by the U.S. census bureau county population, and multiplied by 100,000 to get the incidence per 100,000 people (Source: https://govstatus.egov.com/kycovid19).

The need for physicians has been further exacerbated by the COVID-19 pandemic in the state. In Kentucky, 198,065 cases of COVID-19 were recorded and 2,062 deaths have been reported by early December 2020 (Figure 2). COVID-19 cases are reported in every single county in the state [8].

The pandemic amplified the additional need for physicians in the “hot spots” or in rural hospitals that were already short of physician coverage. With 61.5% of the physicians in the sample practicing in a “frontline” COVID-19 pandemic specialty and 92.3% of them being involved in direct care of COVID-19 patients, they do provide invaluable care to the needs of the state. Furthermore, 100% of these physicians were board-certified in their respective specialties, and a significant part of them were also involved in administrative responsibilities of the COVID-19 preparedness in their hospitals. Our results show that almost 50% of these physicians were approached by a recruiter or a hospital to provide part-time services in COVID-19 designated hot spots, but 92.3% of them reported that visa-related restrictions limited them from doing so. The visa restrictions also limited 53.8% of them from providing telemedicine services. Immigrant physicians have no flexibility to travel to areas to provide services where they are needed the most during this pandemic due to employer-based visa restrictions.

While not being able to provide services in the time when they are most needed, the insecurities around immigration policies also add further challenges for these physicians and their families at a personal front. As illustrated in Table 3, 88.9% of the physicians in the sample expressed the concern that their families could face hardship due to their unemployment or disability during the pandemic, and 66.7% of them were worried that their families are at risk of deportation in case of disability or death of the physician, which is highly likely during the pandemic. Irrespective of the pandemic status, “aging out” phenomenon, where the children of IMG physicians on a visa are at risk of deportation at the age of 21, has been a long-lasting concern [18]. Around 7.1% of the physicians in our sample might face this crisis if the immigration backlog is not rectified in the coming few years. No wonder, 88.5% of the physicians did express that a permanent residency status will solve most of their concerns and challenges.

Healthcare disparities whether they are based on gender, race, ethnicity, or geography, have foiled our healthcare system at multiple levels [19-21]. At the same time, the challenges faced by 24% of our physician workforce (IMG physicians) further hamper the care of the underserved and rural communities like the ones in Kentucky [22].

Expansion of graduate medical education programs, physician loan waiver programs, and dedicated rural medicine tracks are some proposed solutions to improve physician supply to the rural states of Kentucky [23]. At the same time, a few federal immigration legislations have helped solve the aforementioned issues of the IMGs and also improved physician access to the rural communities [24,25]. The “Healthcare Workforce Resilience Act (S.3599)” was introduced in April 2020 to bolster the state and nation’s physician workforce in the COVID-19 crisis, which recaptures the unused visas from previous fiscal years for doctors and nurses and exempt these visas from country caps [24]. Expansion of the Conrad 30 J-1 Visa Waiver Program (S.948) allows states like Kentucky to retain and recruit 35 IMGs into their rural states rather than the existing 30 and grants them permanent residency at the end of five years of service in a medically underserved area [25].

Our survey is limited by the timing of the study as it was conducted in April 2020, when the COVID-19 pandemic was in the early stages in the US and in Kentucky; we anticipate that the hardships of physicians might have worsened in the subsequent months.

## Conclusions

The rural state of Kentucky is burdened by multiple chronic medical problems including physician shortage, and the healthcare needs are further exacerbated during the COVID-19 pandemic. IMG physicians continue to play a valuable role in taking care of the underserved population of the state and fighting the COVID-19 pandemic. The challenges surrounding the immigration policies, especially immigration backlog, contribute to significant hardships for these physicians and their families. At the same time, these policies also remain as a hurdle to expand healthcare access to the medically underserved communities.
